# Therapeutic principles and unmet needs in the treatment of cough in pediatric patients: review and expert survey

**DOI:** 10.1186/s12887-022-03814-0

**Published:** 2023-01-21

**Authors:** Christian Vogelberg, Francisco Cuevas Schacht, Christopher P. Watling, Laura Upstone, Georg Seifert

**Affiliations:** 1 Paediatric Department, University Hospital Carl Gustav Carus Dresden, Technical University Dresden, Dresden, Germany; 2 Department of Pulmonology and Thoracic Surgery, National Institute of Paediatrics, Mexico City, Mexico; 3 Cambridge – a Prime Global Agency, Cambridge, UK; 4 Department of Paediatric Oncology/Haematology, Otto-Heubner Centre for Paediatric and Adolescent Medicine (OHC), Charité – Universitätsmedizin Berlin, Corporate Member of Freie Universität Berlin, Humboldt-Universität zu Berlin, and Berlin Institute of Health, Berlin, Germany

**Keywords:** Cough, Child, Pediatric, Guidelines, Humans, Surveys and questionnaires, Internationality

## Abstract

**Background:**

There are evidence gaps in the management of pediatric cough, particularly for acute pediatric cough. This study had two aims: to identify therapeutic principles and unmet needs in the treatment of cough in pediatric patients (internationally), and to consider the evidence required to address these unmet needs.

**Methods:**

A MEDLINE/PubMed database search was performed to identify articles describing therapeutic principles in the treatment of pediatric cough. An online survey of international pediatric cough experts was conducted, with questions on the definitions, diagnosis, treatment, and unmet needs in pediatric cough management.

**Results:**

Cough guidelines have differing definitions of pediatric patients (≤12–18 years), acute pediatric cough (< 2–3 weeks), and chronic pediatric cough (> 4–8 weeks). Similarly, among 18 experts surveyed, definitions varied for pediatric patients (≤10–21 years), acute pediatric cough (< 3–5 days to < 6 weeks), and chronic pediatric cough (> 2–8 weeks). Guidelines generally do not recommend over-the-counter or prescription cough medicines in acute pediatric cough, due to lack of evidence. In the expert survey, participants had differing opinions on which medicines were most suitable for treating acute pediatric cough, and noted that effective treatments are lacking for cough-related pain and sleep disruption. Overall, guidelines and experts agreed that chronic pediatric cough requires diagnostic investigations to identify the underlying cough-causing disease and thereby to guide treatment. There are unmet needs for new effective and safe treatments for acute pediatric cough, and for randomized controlled trials of existing treatments. Safety is a particular concern in this vulnerable patient population. There is also a need for better understanding of the causes, phenotypes, and prevalence of pediatric cough, and how this relates to its diagnosis and treatment.

**Conclusions:**

Whereas pediatric cough guidelines largely align with regard to the diagnosis and treatment of chronic cough, there is limited evidence-based guidance for the management of acute cough. There is a need for harmonization of pediatric cough management, and the development of standard guidelines suitable for all regions and patient circumstances.

**Supplementary Information:**

The online version contains supplementary material available at 10.1186/s12887-022-03814-0.

## Background

Cough is a highly prevalent symptom in children of all ages and in different regions of the world [1, 2]. Pediatric cough is most commonly caused by acute viral respiratory tract infection, though it can be triggered by numerous conditions including bacterial infection, allergic response, and asthma [3]. Pediatric cough has a major impact on sleep, school performance, and the ability to play and, therefore, has a negative influence on children’s quality of life [3–5]. Pediatric cough is also a source of stress and anxiety for parents, due to its impact on children’s behavior (e.g., refusing food) and sleep patterns, and the often unwarranted fear that it will escalate into a more serious health condition [6, 7]. Thus, pediatric cough is a major driver to seek medical attention, and is associated with high costs resulting from physician visits, diagnostic tests, and medications, thereby placing a high burden on healthcare systems [7–12].

Various local, national, and international organizations have developed clinical practice guidelines in order to standardize the treatment of cough in adults and/or children [12, 13]. However, these guidelines vary in quality, and recommendations are often based on low-quality evidence (observational studies or expert consensus, as opposed to randomized controlled trials [RCTs]) [13]. Cough is a symptom of multiple diseases and, therefore, is managed by a variety of disciplines [12]. Furthermore, while guidelines for adults outnumber those for children, adult guidelines do not translate well to children [13, 14]. Overall, there are evidence gaps in the management of pediatric cough, particularly for acute pediatric cough. The aims of this study were to identify and present therapeutic principles and unmet needs in the treatment of cough in pediatric patients, at an international level, and to consider the clinical studies required to address these unmet needs. The aims are addressed through a review of current literature on pediatric cough, and expert opinion collected using a questionnaire.

## Methods

### Literature review search strategy

A literature review was conducted to search for articles describing therapeutic principles in the treatment of cough in pediatric patients. The review comprised a search of the MEDLINE/PubMed database, supported by relevant articles that were referenced within search results (i.e., primary sources) and by additional articles known to the authors. The following search terms were used: cough [title] AND (pediatric or children) AND treatment. The literature database search was limited to articles/studies in humans, and restricted to articles in the English language published in the previous 10 years. The search was performed on March 13, 2020.

### Questionnaire

A 30-minute online questionnaire was designed by CV, CPW, LU and GS (and reviewed by the study sponsor) to answer the following research question: “According to international experts, what are the therapeutic principles for, and unmet needs in, the treatment of cough in pediatric patients?” The questionnaire is provided as Additional file 1.

The target audience for the questionnaire was a minimum of 20 international healthcare professionals with expertise in the area of pediatric cough. Initially, the names and email addresses of 105 candidates to receive the questionnaire were identified by a process of expert mapping using publicly available information, based on publication/guideline authorship, editorial board membership, conference participation, and involvement in clinical trials. However, due to a lower than anticipated response rate, the names and email addresses of an additional 217 candidates to receive the questionnaire were provided by the study sponsor, via their international partners. Candidates were sent an introductory email describing the questionnaire and inviting them to participate, with follow-up email reminders at 2-weekly intervals. No monetary or other incentive was offered for completing the survey. The survey was intended to run for 4 weeks; in order to counter the low response rate, this became two separate 4-week periods.

The questionnaire comprised an introductory section, in which the nature and purpose of the survey was described, and informed consent was obtained, followed by a section to characterize the medical background of the participants and to determine if they were eligible to complete the survey. Eligibility criteria were as follows: 1) has treated pediatric cough for ≥5 years; and 2) has managed ≥30 pediatric patients with cough in the past 6 months; and 3) in the past 10 years, has either a) spent > 50% of their professional time in clinical practice (as opposed to in an academic or research setting), and/or b) participated in a specialist pediatric cough congress or session within a pediatric respiratory conference (whether in a panel, as a speaker, or as a chairperson), and/or c) been an author on an article relating to pediatric cough published in a peer-reviewed journal, and/or d) worked on national or international pediatric cough guidelines. Eligible participants continued to the remainder of the questionnaire, which comprised a section on the definitions, diagnosis and treatment of pediatric cough, followed by a section on unmet needs in pediatric cough. A mix of open- and closed-ended questions were used.

The questionnaire was built using SurveyMonkey (Momentive Inc., San Mateo, CA). Data were analyzed using Excel (Microsoft Corporation, Redmond, WA). For quantitative questions, means and numbers/percentages were calculated, as appropriate. No statistical comparisons were conducted due to the descriptive nature of the study and the small sample size.

## Results

### Definitions, diagnostic and therapeutic principles, and unmet needs in pediatric cough – literature review

In the literature review, key pediatric cough guidelines were identified from the American College of Chest Physicians (CHEST) [15], an Australian multidisciplinary expert committee [16], the British Thoracic Society (BTS) [3], and the European Respiratory Society (ERS) [17]. These guidelines are summarized below, supplemented by relevant information from clinical studies and reviews. Of note, the definition of pediatric patients varies among cough guidelines, from up to 12–18 years (and in some cases is undefined) [3, 15–19]. All guidelines separated cough into acute and chronic, and most guidelines focused on chronic cough.

### Acute pediatric cough

#### Definitions, causes, and diagnosis of acute cough

Acute pediatric cough is defined as a cough lasting for < 2–3 weeks, depending on the guideline [3, 16]. Certain guidelines also define subacute or prolonged acute cough to describe ‘the gray area’ between acute and chronic cough, when symptoms may be slowly resolving (for example, for patients with pertussis or post-viral cough) [3, 16].

Acute cough is most commonly caused by a viral upper respiratory tract infection (URTI) [3]. Other possibilities include an inhaled foreign body, seasonal allergic rhinitis, or the first presentation of a chronic disease [3]. Acute pediatric cough caused by URTI can be diagnosed from patient history and physical examination, and generally does not require further investigation [3, 20, 21].

#### Treatment of acute cough

Cough caused by URTI normally resolves spontaneously, without treatment [20, 21]. Parents may require education and reassurance that cough will subside given time [3, 21].

There is no good evidence for or against the effectiveness of over-the-counter (OTC) medicines in acute cough (including antitussives, expectorants, mucolytics, and antihistamines) and, consequently, these are not recommended by pediatric treatment guidelines [3, 15, 18, 22]. The safety of OTC medicines in children has been questioned; however, the overall adverse event rate appears to be low [22, 23]. Though generally not mentioned in pediatric cough guidelines, certain phytomedicines have shown efficacy on acute cough severity in pediatric patients, with few adverse events [24]. There is some evidence that honey is more effective than placebo in relieving acute cough in children [18, 25]. However, honey is not recommended in very young children (< 1 year) due to a risk of botulism [18, 26, 27]. Antibiotics are not effective or recommended for treating URTIs [3, 28]. Bronchodilators are not effective for acute cough in children without asthma (asthma treatment is described below) [3].

### Chronic pediatric cough

#### Definitions, causes, and diagnosis of chronic cough

Chronic pediatric cough is generally defined as a cough lasting for > 4 weeks [15–17]. The exception is the BTS, which defines chronic pediatric cough as lasting for > 8 weeks, while acknowledging that there is a gray area between acute and chronic, and that ‘relentlessly progressive’ cough should be investigated after 3 weeks [3]. Certain pediatric cough guidelines recommend characterizing chronic cough into wet/productive or dry, because this distinction affects diagnosis and treatment pathways, with wet cough often having an infectious etiology [3, 15, 17, 19, 20]. Guidelines also make the distinction between ‘specific’ cough, which is associated with a condition recognized to cause cough, thereby forming the basis for specific treatment, and ‘non-specific’ cough, in which no underlying condition has yet been found [3, 15–17, 21].

The most common causes of chronic pediatric cough are generally thought to be protracted bacterial bronchitis (PBB), asthma, and post-infectious cough [10, 16, 17, 29, 30]. In a study of children with acute respiratory illness and cough presenting at an emergency department, 20.4% had persistent cough 4 weeks later; 6.6% were subsequently diagnosed with PBB, and 4.3% with a new chronic respiratory disease [31]. PBB is most common in children aged < 6 years [32], and, in many cases, onset of symptoms occurs before the age of 1 year [33]. Less common, but more serious, are lung disorders (such as cystic fibrosis) and immune deficiency [3, 15–17]. Habit cough is a repetitive, chronic cough with no identified underlying organic reason; habit cough is more common in older children, peaking at around 10 years of age [34–37]. Chronic cough may also be caused or exacerbated by exposure to airborne irritants, such as tobacco smoke, allergens, and traffic pollution [3, 15–17, 21].

With regard to diagnosis, chronic cough in children should be seen as a symptom of an underlying disease and, therefore, should be subject to careful and systematic evaluation for the presence of specific diagnostic indicators [3, 15–17, 19, 38]. Diagnosis should involve a detailed patient history, thorough physical examination, chest X-ray, and, if the child is old enough and cooperative, spirometry [3, 15–17, 19, 20]. Sputum cultures should be attempted in cases of wet chronic cough; further investigations (such as bronchoscopy and serological laboratory assessments) may be required, after giving consideration to patient discomfort and the potential for adverse events [3, 15, 17, 19, 20]. Habit cough can be diagnosed from clinical characteristics; the key diagnostic feature is that coughing stops when the child is asleep [3, 17, 21, 36, 39].

#### Treatment of chronic cough

The management of specific chronic pediatric cough should be based on the etiology of the cough, as symptoms will resolve if the underlying condition is successfully managed [3, 15, 17, 19]. For example, children with PBB should receive antibiotics, and children with asthma may benefit from inhaled bronchodilators and inhaled corticosteroids [3, 16, 21].

For non-specific chronic pediatric dry cough, a treatment attempt with inhaled corticosteroids may be appropriate; for non-specific chronic wet/productive cough, antibiotics may be trialed [15, 17, 20, 40]. While antibiotics are considered efficacious in the treatment of children with chronic wet cough, the indication for antibiotics should be checked to avoid unnecessary exposure to possible side effects such as vomiting, diarrhea, and rash [41], and antibiotic resistance. For children exposed to airborne irritants such as tobacco smoke, allergens, or home pollutants, attempts should be made to remove the child from this environment (i.e., stopping the child and/or their parents from smoking) [3, 15–17].

Numerous algorithms are available to assist in the treatment of chronic pediatric cough [3, 15, 17, 20, 42], and the use of such algorithms improves clinical outcomes [43].

### Unmet needs in pediatric cough

Several articles in the literature review identified unmet needs in the management of pediatric cough. In terms of treatment, there is a dearth of evidence from adequately performed RCTs for the efficacy of current OTC and prescription products for acute cough [13, 22, 44]. RCTs are also needed to assess treatment efficacy in different clinical settings, such as community versus hospital, and among different regions of the world [19]. Overall, the number of therapeutic options for acute cough is limited, and there is a need for new medications that can suppress acute cough and relieve patient distress, without side effects [44].

Although treatment guidelines improve clinical outcomes in chronic cough, they are often based on low-quality evidence [13, 43, 45]. Treatment algorithms have not been tested to see if children have different needs based on the duration and/or severity of their cough [19]. Furthermore, the most appropriate age cutoff for the use of pediatric versus adult cough guidelines has not been determined [19].

Unmet needs also relate to the etiology and progression of pediatric cough. Studies are required to explore the progression of acute cough to chronic cough (current evidence suggests that clinical review is warranted when chronic cough develops following acute respiratory illness in children [31]), the progression of chronic cough with time, and to determine the factors that predict this progression [17, 19, 46]. At present, when a patient’s cough does not respond to standard therapy, it can be unclear if the treatment was unsuitable, or if the etiology of cough was incorrectly discerned [44]. There are also questions relating to the overlap of chronic cough with other conditions, such as respiratory disease [17]. With regard to PBB, although initial research has looked into its risk factors and progression [47], further research is needed on its natural history, underlying disease mechanisms (factors influencing impaired pathogen clearance mechanisms), and how to optimize its treatment [48].

Finally, there is a need for additional studies on the clinical and psychosocial impact of cough on children and their families, and on the economic burden of cough to the individual and society [17, 46].

### Definitions, diagnostic and therapeutic principles, and unmet needs in pediatric cough – expert survey

The expert survey ran from November 23 to December 21, 2020 (105 candidates identified from expert mapping) and May 24 to June 21, 2021 (217 candidates provided by the study sponsor). In total, 322 email invitations were sent out, of which 19 emails were undelivered.

### Participant characteristics

Thirty healthcare professionals (9.9%) gave consent to participate in the survey; however, one participant did not answer any questions after giving consent. Participants represented 16 countries across Asia (Israel, Malaysia, Qatar, United Arab Emirates), Australia, Europe (Austria, Bosnia and Herzegovina, Croatia, Germany, Italy, Serbia, Slovenia, United Kingdom), North America (Dominican Republic, United States), and South America (Bolivia).

The most common primary medical specialty was general pediatrics (20/29 participants; 69%); pediatric allergy, pulmonary, and respiratory specialties were also represented. All 29 participants had been treating pediatric cough for ≥5 years, and 26 participants (90%) had > 10 years of experience. Twenty-five participants (86%) had managed ≥30 pediatric patients with cough in the past 6 months, of whom 14 (48%) had managed > 100 such patients; however, 3 participants (10%) had managed < 30 patients and were ineligible to continue, as was 1 participant (3%) who did not answer the question.

Regarding the expertise of participants, 15/25 (60%) spent > 50% of their professional time in clinical practice, 13 (52%) had participated in a specialist pediatric cough congress or session within a pediatric respiratory conference, 8 (32%) had been an author on an article relating to pediatric cough published in a peer-reviewed journal, and 9 (36%) had worked on national or international pediatric cough guidelines. However, 5 participants (20%) did not meet any of these criteria and 2 (8%) did not answer the question, meaning that a total of 18 participants were eligible to proceed to the next section of the survey.

### Definitions and diagnosis of pediatric cough

**Fig. 1:**
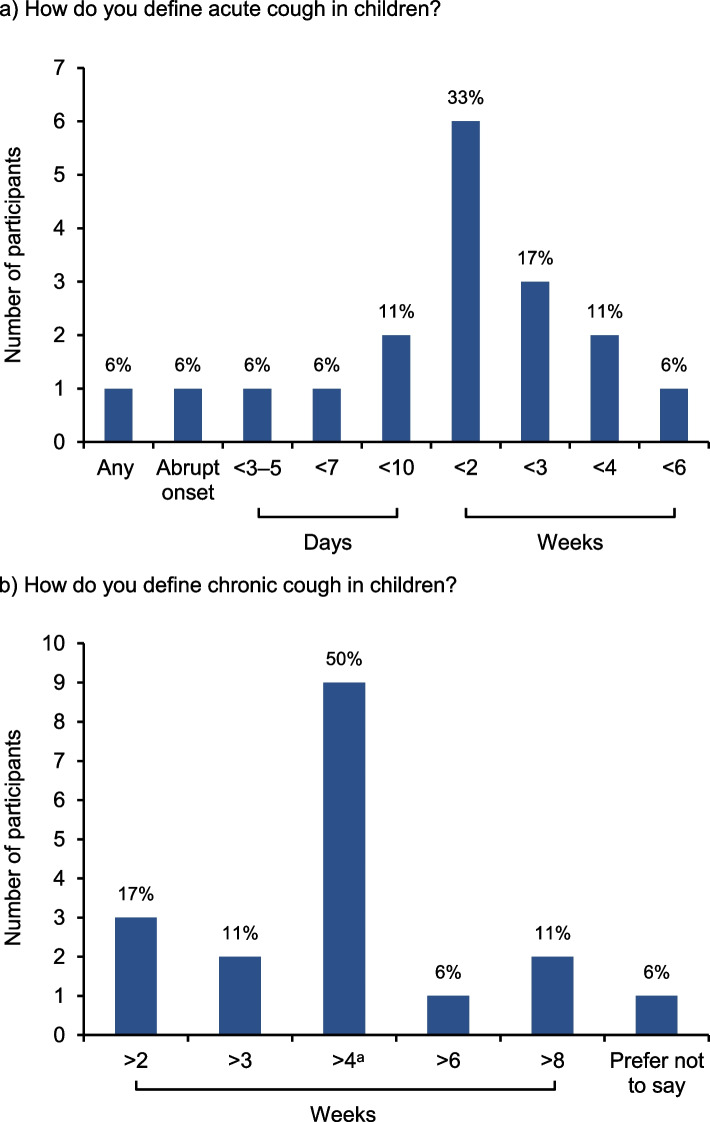
Definitions of acute and chronic pediatric cough according to international experts (*n* = 18). ^a^Or > 30 days. Questions were open-ended

Participants suggested a range of ages to define ‘pediatric’ cough, most commonly ≤18 years (9/18; 50%) or ≤ 16 years (3/18; 17%), with ≤10, ≤12, ≤15, ≤17, and ≤ 21 years suggested by 1 participant each (and 1 participant preferred not to say). Definitions provided for acute cough in children ranged from < 3–5 days to < 6 weeks (or no specified duration), most commonly < 2 weeks (Fig. 1a). Definitions for chronic cough in children ranged from > 2 weeks to > 8 weeks, most commonly > 4 weeks/> 30 days (Fig. 1b).

Almost all participants (17/18; 94%) said that they distinguish between wet/productive and dry cough, for reasons including to help determine the cause and diagnosis, and to inform cough management. One participant said that they do not make this differentiation, since “it is not associated with the cause” of cough.

Regarding clinical guidelines, 11/18 participants (61%) said that their country had national clinical guidelines for the treatment of cough in adults, 12 (67%) said that their country had national clinical guidelines for the treatment of cough in pediatrics, 4 (22%) had no national guidelines, and 1 (6%) did not know if their country had guidelines. Ten of 17 participants (59%) said that they used a guideline for the diagnosis of pediatric cough, and 12 (71%) said that they used a guideline for the treatment of pediatric cough. Specific guidelines were named from the following organizations and countries: American Academy of Pediatrics; American Thoracic Society; Australia’s guideline; Austria’s guideline; BTS; Centers for Disease Control and Prevention; ERS (cough and protracted bacterial bronchitis); Germany’s national guideline; Indian Academy of Pediatrics; Malaysia’s guideline; National Institute for Health and Care Excellence; Serbia’s national guideline; Slovenia’s “cough in children” guideline; and two reviews from the United States [49, 50].

When participants were asked under what circumstances they perform further diagnostic investigations concerning the cause of pediatric cough (beyond patient history and physical examination), the most common response was in patients with “chronic cough” (10/16 participants; 63%), particularly if not responding to usual treatments, for repeated episodes, or in the presence of other disease symptoms. Other conditions spontaneously mentioned by one or more participants that would warrant further diagnostic investigations were allergy, aspiration/inhaled foreign body, chronic pulmonary disease, cyanosis, newborns coughing during feeding, pertussis, respiratory distress, and severe infection/pneumonia. When asked to indicate the approximate percentage of their pediatric patients with cough in which specific investigations were instigated in the past 6 months, median percentages were 31–40% for spirometry, 21–30% for chest X-ray and allergy testing, 11–20% for sputum culture, 1–10% for serology and computerized tomography scan, and 0–10% for bronchoscopy. One participant noted that fewer children with cough were referred during the COVID-19 pandemic, and no pulmonary function tests were carried out due to the potential for aerosol generation.

### Treatment of pediatric cough

When asked on what single aspect does your choice of treatment for pediatric cough primarily depend, 10/15 participants (67%) said “the cause of cough”, and 5 (33%) said “symptoms (e.g., cough frequency, intensity)”. Three participants (20%) said that they treat acute cough immediately upon presentation, 7 (47%) said that they treat after watching and waiting to see if the cough resolves by itself, and 5 (33%) said it depends on specific circumstances.

When participants were asked if there are any treatment traditions for pediatric cough specific to their country/region, 11/17 (65%) said that there were traditions; however, few participants said that they actually followed the traditions (5/17; 29%). Examples given for local treatment traditions that are followed were: cough syrup/inhalation therapy (Germany), a period of observation (Slovenia), and herbal medicine/cough syrup/honey (United Arab Emirates). Examples given for local treatment traditions that are not followed were: antitussives/expectorants in infants with obstructive bronchitis (Croatia), inhalation therapy (Italy), guava leaves (Qatar), herbal medicines (Serbia), and anti-asthma therapy for problem cough without investigating diagnosis (United Kingdom).

**Fig. 2:**
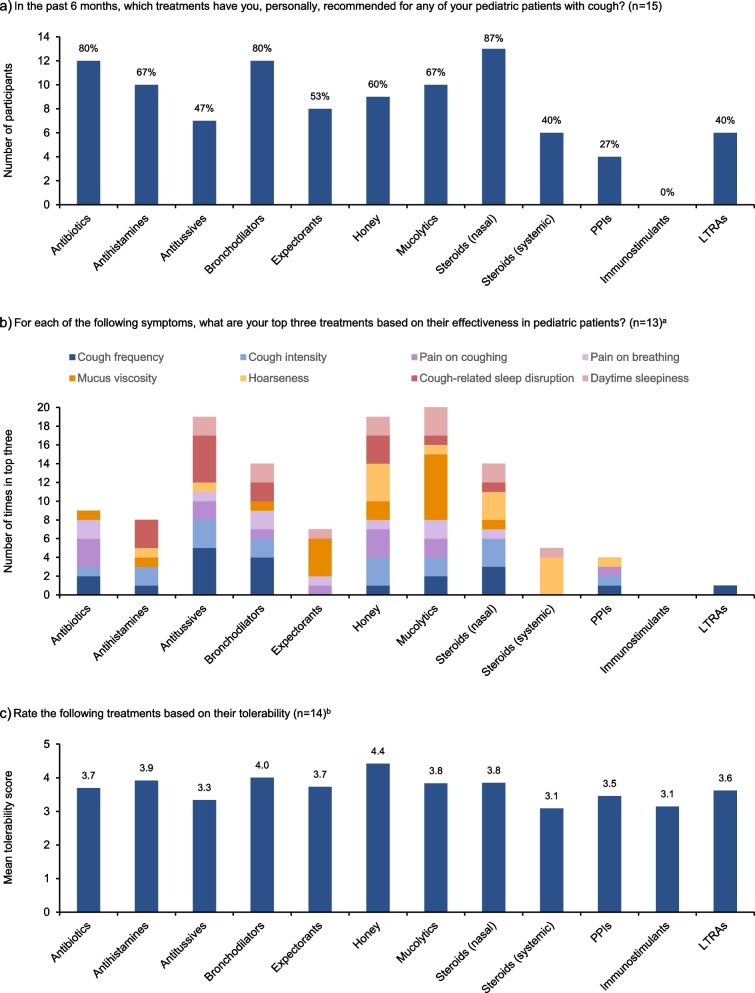
Utilization, efficacy, and tolerability of current treatments for pediatric cough according to international experts. ^a^Answers of “None” and “Prefer not to say” are not shown. If a participant selected the same treatment multiple times for a particular symptom, it was counted once only. ^b^Participants rated the tolerability of each treatment as very good (5), good (4), moderate (3), poor (2), or very poor (1). Answers of “Prefer not to say” are not shown. Questions were closed-ended. LTRA, leukotriene receptor antagonist; PPI, proton pump inhibitor

In the past 6 months, nasal/inhaled steroids, antibiotics, and bronchodilators had been recommended by ≥80% of participants for pediatric patients with cough (Fig. 2a). Antibiotics were generally recommended when cough had a bacterial cause. The majority of participants (12/15; 80%) had administered some form of combination therapy, with the specific combination depending on patient circumstances. When asked specifically about phytomedicines, 11/15 participants (73%) recommended their use in pediatric patients, with ivy leaf extract being the most commonly recommended phytomedicine (10/15; 67%).

To assess treatment effectiveness, participants were asked to rank their top three treatments for various symptoms (Fig. 2b). Antitussives and bronchodilators were most commonly picked in the top three for cough frequency; mucolytics and expectorants were most commonly picked in the top three for mucus viscosity; and antitussives were most commonly picked in the top three for cough-related sleep disruption. Across all symptoms, mucolytics, antitussives, and honey were most commonly ranked in the top three treatments. Of note, the answer ‘none’ was selected in the top three by ≥3 participants for cough intensity, pain on coughing, pain on breathing, cough-related sleep disruption, and daytime sleepiness. In general, participants said that they assess treatment effectiveness in their clinics based on improvement or resolution of symptoms (e.g., cough frequency and intensity, sputum production), and effects on quality of life and patient satisfaction.

Treatments rated as the most well tolerated were honey, bronchodilators, and antihistamines; treatments rated the least well tolerated were systemic steroids, immunostimulants, and antitussives (Fig. 2c).

### Unmet needs in pediatric cough

The greatest unmet needs in pediatric cough, as entered into an open-ended text box by 14 experts, are presented in Table 1. Themes included the need for effective and safe new treatments, better understanding of causes and phenotypes of cough, and education on the prevention of respiratory infection.

**Table 1:** Unmet needs and data gaps in pediatric cough according to international experts

**What, in your opinion, are the greatest unmet needs in pediatric cough? (*n* = 14)**	
**Treatment**	**Etiology and progression**
Effective, safe treatment for acute viral cough	Understanding causes
Faster improvement/resolution of cough symptoms	Understanding different cough phenotypes and their treatment
Cough medication for children aged < 2 years	**Other** Education in a preschool setting on the prevention and management of respiratory infection Allergy [unspecified] Clinical studies in pediatric populations [unspecified]
Effective, safe treatment for acute cough that interferes with sleep	
Mucolytics for dry cough	
Specific cough suppressants	
Testing and treatment for suspected recurrent viral bronchitis	
**What data gaps are there in pediatric cough research that you would like to see addressed in a clinical study? (*n* = 12)**	
**Treatment**	**Etiology and progression**
Controlled clinical trial on suggestion therapy for habit cough	Determining cough phenotypes in different age groups
Effective treatment of viral cough	Is wet/dry cough accurately reported, and does it change over time?
Effectiveness of over-the-counter drugs	Main sources of respiratory infection in preschool children
Efficacy and safety of symptomatic cough drugs (e.g., secretolytics, mucolytics, antitussives, and protussives)	Prevalence of chronic cough with accurate assessment of causes
Safety and effective dosing of drugs in children aged < 2 years	Prevalence of pertussis causing prolonged cough
Study of herbal versus allopathic chemicals	Cough receptor sensitivity in children
Testing and treatment for suspected recurrent viral bronchitis	**Other** Advanced allergic tests Rapid diagnosis of pertussis in patients with prolonged cough National guidelines [unspecified]

Questions were open-ended, and answers were grouped into ‘Treatment’, ‘Etiology and progression’, and ‘Other’ during analysis

Finally, participants were asked the open-ended question, “Which data gaps in pediatric cough research would you like to see addressed in a clinical study?” (Table 1). Several participants suggested efficacy and safety studies to establish the benefits of existing cough treatments in pediatric patients (e.g., OTC drugs, herbal therapies). Other participants suggested efficacy and safety studies in subpopulations of pediatric cough (e.g., aged < 2 years, viral cough, habit cough, viral bronchitis). In addition, several participants suggested prevalence studies for particular types of pediatric cough (e.g., chronic, wet/dry, pertussis).

## Discussion

This study, comprising a literature review together with expert opinion obtained via an online survey, has highlighted a number of issues relating to the global management of pediatric cough. In particular, while there are numerous regional guidelines for the management of pediatric cough (16 were listed in the survey), there is no established international guideline for acute or chronic pediatric cough.

There is also a lack of consensus as to what defines a pediatric patient in relation to cough. In guidelines, ‘pediatric’ refers to patients aged up to 12–18 years, and among the international experts, ‘pediatric’ could refer to patients aged up to 10–21 years. The age of patients is an important consideration in their management as it determines whether pediatric or adult guidelines are used, impacting pediatric-specific issues and risk–benefit ratios [15, 19]. For example, whereas respiratory function tests are standard investigations in adults, data generated from such tests may be unreliable in young children [19]. The optimal management of cough may also vary between babies, toddlers, and older children due to differing cough etiologies with age; a point that is not fully addressed by current guidelines. Furthermore, while all guidelines separated pediatric cough into acute and chronic, there were discrepancies in the duration of cough used to define these terms. Acute cough definitions ranged from < 2–3 weeks in guidelines and from < 3–5 days to < 6 weeks among experts; the equivalent definitions for chronic cough were > 4–8 weeks and > 2–8 weeks. Since acute and chronic cough are managed differently, these varying definitions mean that pediatric patients in different regions will receive differing standards of care for cough.

Regarding diagnosis, guidelines agreed that acute pediatric cough (which is most commonly URTI-based) can usually be diagnosed by patient history and examination, whereas chronic pediatric cough requires systematic evaluation to identify the underlying disease. Most experts agreed that chronic pediatric cough requires further diagnostic investigations, and each expert listed a variety of investigations they had used in the past 6 months (most commonly spirometry, chest X-ray, and allergy testing), presumably representative of the patients that had presented at their respective clinics, and the testing that was possible during the COVID-19 pandemic.

Considering the treatment of acute cough symptoms, guidelines highlighted the lack of evidence for OTC and prescription cough medicines. This was mirrored in the expert survey, where participants had differing opinions on which medicines were most suitable for different symptoms. Experts considered honey to be one of the most effective and well tolerated acute cough treatments, with some support from clinical studies [25]. Most experts had recommended phytomedicines for the treatment of acute pediatric cough. Cough-related pain and sleep disruption were highlighted as symptoms for which current therapies are lacking. The majority of children with cough (and their parents) suffer from disturbed sleep [4], and there is a need for additional treatment options in this area. Experts judged that systemic steroids and immunostimulants are generally not efficacious, and are among the least well tolerated options in children, indicating a need for alternative rapid-acting treatment options. Considering chronic cough, there is greater consensus on its treatment, which involves addressing the underlying cough-causing disease.

Across the literature review and expert survey, various unmet needs and data gaps were identified in the management of pediatric cough. The most frequently reported need was for effective and safe treatments for acute cough – both the development of new treatments, and obtaining strong RCT evidence for existing treatments. The safety of current treatments at different doses is a particular concern in this vulnerable patient population. There is also a need for better understanding of the causes, phenotypes, and prevalence of pediatric cough, and how this relates to diagnosis and treatment, remembering that cough itself is not a disease, and can be viewed from a variety of perspectives including as a defensive reflex and a symptom.

Several challenges are associated with conducting clinical trials in pediatric cough. Acute cough associated with URTI is typically transient and self-limiting, meaning that a large trial or a large effect size is necessary to demonstrate a clinically relevant effect [44]. There is a seemingly large response to placebo in clinical trials of acute cough therapies (up to 85%) – attributed to natural recovery, the physiological stimulation of saliva and mucus, as well as psychological effects (the true placebo effect) – meaning that it is difficult to demonstrate the superiority of active treatment versus placebo [51, 52]. There is a need for validated, automated, real-time cough counting technology, to allow the objective assessment of clinical response [17, 53]. In addition, pediatric patient-reported outcomes for cough, such as cough-specific quality of life, need to be developed and validated [17, 54].

The present study was limited by the small sample size of the survey, which is, in part, due to the strict entry criteria, designed to identify top experts in the field of pediatric cough. The survey coincided with the COVID-19 pandemic, which may have reduced the availability of respiratory disease experts. Although five continents were represented, there was a bias towards European doctors, and not all regions were represented (e.g., Africa). The survey had a relatively low response rate (9.9%), and was conducted over two time periods. While expert opinion is valuable, it may not be representative of day-to-day clinical practice, and is not a substitute for clinical evidence. The survey was designed prior to, but conducted during, the COVID-19 pandemic, which may have influenced participant answers to questions, particularly those intended to obtain information on their recent approach to diagnosis and management. Finally, the literature database search was limited to the previous 10 years in order to increase the efficiency of the search and to obtain the most recent updates to guidelines; it is possible that guidelines > 10 years old were missed, despite being current.

## Conclusions

The management of pediatric cough is not standardized across the world. International experts use a variety of different guidelines, and have diverse opinions on how to define aspects of pediatric cough. Whereas pediatric cough guidelines largely align with regard to the diagnosis and treatment of chronic cough, there is limited evidence-based guidance for the management of acute cough. From an international perspective, there is a need for harmonization of pediatric cough management, and the development of standard guidelines suitable for all regions and patient circumstances. However, the development of such guidelines will require data gaps to be filled. In particular, clinical studies are needed to clarify the efficacy and safety of current acute cough therapies, and to better characterize subpopulations of pediatric patients with cough.

## Supplementary Information

**Additional file 1.** A copy of the survey.

**Additional file 2.** Survey answers dataset.

## Data Availability

The dataset supporting the conclusions of this article is included within the article and its additional files (see Additional file 2).

## Abbreviations

BTS: British Thoracic Society
CHEST: American College of Chest Physicians
ERS: European Respiratory Society
OTC: over-the-counter
PBB: protracted bacterial bronchitis
RCT: randomized controlled trial
URTI: upper respiratory tract infection
