# Supplementary material for: Therapeutic principles and unmet needs in the treatment of cough in pediatric patients: review and expert survey
Source: BMC Pediatr. 2023 Jan 21;23:34. doi: 10.1186/s12887-022-03814-0 (PMC9860236; doi:10.1186/s12887-022-03814-0)
Supplement: Supplementary file 1 — Additional file 1. A copy of the survey. [file 12887_2022_3814_MOESM1_ESM.pdf]

Additional file 1: A copy of the survey

Supplement to: Vogelberg C, et al. Therapeutic principles and unmet needs in the treatment of cough in pediatric patients: review and expert survey.

1. Introduction

Thank you for your interest in taking part in this survey.

The aim of the survey is to answer the following research question:

According to international experts, what are the therapeutic principles for, and unmet needs in, the treatment of cough in pediatric patients?

As well as general treatment principles, the questionnaire includes a section on your experiences with Prospan® ivy leaf extract for the treatment of pediatric cough.

We intend to publish the results of the survey in a peer-reviewed scientific journal within two integrated review papers. You have been invited to take part because you are a healthcare professional with experience in the area of pediatric cough. We hope that approximately 20 healthcare professionals will participate.

The survey is being conducted by Cambridge, a Prime Global Agency (Cambridge, UK) on behalf of Engelhard Arzneimittel (Niederdorfelden, Germany). Your contact information and any information that you provide via the survey will only be utilized for the specific purpose of this survey. This information will be retained securely and in confidence by Cambridge in accordance with the Data Protection Act and General Data Protection Regulations under UK law. Cambridge will ensure that your contact details and all identifiable information you provide is deleted from their systems no later than 3 months after the publication of the manuscripts.

The survey is being conducted in accordance with the Market Research Society (MRS) Code of Conduct. The identity of respondents is confidential and none of your details will be passed to any third party without your permission. Any information you disclose will be treated in the strictest confidence and no answers will be attributable to you as an individual. No questions about individual patients will be asked.

Results of the survey will be aggregated to provide an overall picture of the treatment of pediatric cough. The aggregated findings will be used for market research purposes, will be published, and may be shared with third parties working on behalf of the sponsoring pharmaceutical company. The outputs of this research at an aggregated level, and anonymized quotes, may be used by the sponsoring company in a promotional context.

The questionnaire will take approximately 30 minutes to complete, and must be completed in one session. You have the right to withdraw from the research at any time and to withhold information as you see fit.

[Text in square brackets represents coding instructions and will not be visible to the user]

Q1.1

[Ask all]

Do you consent to participate in the survey?

[Select one]

- Yes [go to section 2]
- No [go to section 6]

2. Characteristics of invitees

We will now ask a few questions about your background, to see if you are eligible to participate in the study. If you are eligible, you will move on to the main questionnaire.

Q2.1

[Ask all]

In which country are you based?

[Select one from a drop-down menu.]

- Australia
- Austria
- Belgium
- Brazil
- Canada
- China
- Croatia
- Germany
- India
- Israel
- Italy
- Japan
- Malaysia
- The Netherlands
- New Zealand
- Nigeria
- Saudi Arabia

- Slovenia
- Spain
- UK
- Uruguay
- USA
- Other [additional text box]

Q2.2

[Ask all]

What is your primary medical specialty?

[Open-ended]

Q2.3

[Ask all]

For how many years have you been treating pediatric cough?

[Select one]

- <5
- 5–10
- >10

[≥5 is eligible to proceed]

Q2.4

[Ask all]

Approximately how many pediatric patients with cough did you manage in the past 6 months?

[Select one]

- <30
- 30–60
- 60–100
- >100

[≥30 is eligible to proceed]

Q2.5

[Ask all]

Approximately how many patients aged <2 years with cough did you manage in the past 6 months?

[Select one]

- <30
- 30–60
- 60–100
- >100

Q2.6

[Ask all]

What is the most common cause of pediatric cough in the cases that you have encountered in the past 6 months?

[Select one]

- Viral infection
- Bacterial infection
- Allergic response
- Asthma
- Other (please specify) [additional text box]

Q2.7

[Ask all]

In the past 10 years, do any of these statements apply to you?

A: I spent more than 50% of my professional time in clinical practice (as opposed to in an academic or research setting)

B: I have participated in a specialist pediatric cough congress or session within a pediatric respiratory conference (whether in a panel, as a speaker, or as a chairperson)

C: I have been an author on an article relating to pediatric cough published in a peer-reviewed journal

D: I have worked on national or international pediatric cough guidelines

- No
- Yes (please enter the letters of the statements that apply to you) [additional text box]

[Answering yes is eligible to proceed.]

Q2.8

[Ask all]

Does your country have national clinical guidelines for the treatment of cough?

[Select one]

- Yes, in adults and children

- Yes, in adults only
- Yes, in children only
- No
- Don’t know

Q2.9

[Ask all]

Do you have experience using phytomedicines (i.e., herbal-based traditional medicines) for cough?

Select all that apply.

[Select any number]

- Yes, in research
- Yes, in clinical practice
- Yes, other (please specify) [additional text box]
- No

[Go to section 3 if eligible]

[Go to section 6 if not eligible]

3. Pediatric cough – therapeutic principles

In this section, we will ask about how you diagnose and treat pediatric cough.

Definitions and diagnosis of pediatric cough

Q3.1

[Ask all]

In relation to cough, how do you define pediatric patients?

[Select one and type answer]

- Age ≤ [additional text box] years
- Don’t know
- Prefer not to say

Q3.2

[Ask all]

How do you define acute cough in children?

[Select one and type answer]

- Duration: [additional text box]
- Don’t know
- Prefer not to say

Q3.3

[Ask all]

How do you define chronic cough in children?

[Select one and type answer]

- Duration: [additional text box]
- Don’t know
- Prefer not to say

Q3.4

[Ask all]

During diagnostic work-up, do you differentiate pediatric cough into wet/productive and dry?

[Select one]

- Yes [go to Q3.4b]
- No [go to Q3.4c]
- Don’t know
- Prefer not to say

Q3.4b

[Ask if answered ‘yes’ to Q3.4]

Why do you differentiate pediatric cough into wet/productive and dry?

[Open-ended]

Q3.4c

[Ask if answered ‘no’ to Q3.4]

Why do you not differentiate pediatric cough into wet/productive and dry?

[Open-ended]

Q3.5

[Ask all]

Do you use a clinical guideline for the diagnosis of pediatric cough?

[Select one]

- Yes (please specify which guidelines) [additional text box]
- No
- Don’t know
- Prefer not to say

Q3.6

[Ask all]

In which circumstances do you perform further diagnostic investigations concerning the cause of pediatric cough, beyond patient history and physical examination?

[Open-ended]

Q3.7

[Ask all]

In the past 6 months, which further diagnostic investigations concerning the cause of cough have you instigated for any of your pediatric patients?

Indicate the approximate percentage of patients.

[Select drop-down menu options in 10% increments: 0%, 1–10%, 11–20%, etc.; ‘don’t know’ and ‘prefer not to say’ options will also be included]

- Bronchoscopy
- Chest X-ray
- Spirometry
- Sputum culture
- Serology
- Allergy test
- CT scan
- Other (please specify) [additional text box]

Treatment of pediatric cough

Q3.8

[Ask all]

Do you use a clinical guideline for the treatment of pediatric cough?

[Select one]

- Yes (please specify which guidelines) [additional text box]
- No

- Don’t know
- Prefer not to say

Q3.9

[Ask all]

Are there any treatment traditions for pediatric cough specific to your country/region?

[Select one]

- Yes, and I follow them [go to Q3.9b]
- Yes, but I do not follow them [go to Q3.9b]
- No
- Don’t know
- Prefer not to say

Q3.9b

[Ask if answered ‘yes’ to Q3.9]

Please describe the treatment traditions for pediatric cough specific to your country/region.

[Open-ended]

Q3.10

[Ask all]

In the past 6 months, which treatments have you, personally, recommended for any of your pediatric patients with cough?

Select all that apply.

[Select any number]

- Antibiotics
- Antihistamines
- Antitussives
- Bronchodilators
- Expectorants
- Honey
- Mucolytics
- Steroids (nasal/inhaled)
- Steroids (systemic)
- Proton pump inhibitors
- Immunostimulants

- Leukotriene receptor antagonists
- Other (please specify) [additional text box]
- None
- Prefer not to say

Q3.11

[Ask all]

When do you most commonly start treating acute pediatric cough?

[Select one]

- Immediately upon presentation
- After watching and waiting to see if the cough resolves by itself
- Other (please specify) [additional text box]
- Prefer not to say

Q3.12

[Ask all]

On what aspect does your choice of treatment for pediatric cough primarily depend?

[Select one]

- The cause of cough
- Symptoms (e.g., cough frequency, intensity)
- Other (please specify) [additional text box]
- Prefer not to say

Q3.13

[Ask all]

How do you determine if a treatment for pediatric cough is effective in your patients?

[Open-ended]

Q3.14

[Ask all]

Please pick and rank your top three treatments based on their effectiveness for reducing the following symptoms (i.e., symptomatic treatment) in pediatric patients.

If the third column is not visible, please scroll across using the bar beneath this table.

	Most effective	Second most effective	Third most effective
Cough frequency	[Pick from list]	[Pick from list]	[Pick from list]
Cough intensity	[Pick from list]	[Pick from list]	[Pick from list]
Pain on coughing	[Pick from list]	[Pick from list]	[Pick from list]
Pain on breathing	[Pick from list]	[Pick from list]	[Pick from list]
Mucus viscosity	[Pick from list]	[Pick from list]	[Pick from list]
Hoarseness	[Pick from list]	[Pick from list]	[Pick from list]
Cough-related sleep disruption	[Pick from list]	[Pick from list]	[Pick from list]
Daytime sleepiness	[Pick from list]	[Pick from list]	[Pick from list]

[List of treatments for drop-down menus]

- Antibiotics
- Antihistamines
- Antitussives
- Bronchodilators
- Expectorants
- Honey
- Mucolytics
- Steroids (nasal/inhaled)
- Steroids (systemic)
- Proton pump inhibitors
- Immunostimulants
- Leukotriene receptor antagonists
- None
- Don’t know
- Prefer not to say

Q3.15

[Ask all]

Please rate the following treatments based on their tolerability.

[Select one from a 5-point rating scale ranging from ‘very good’ to ‘very poor’ presented for each category; ‘don’t know’ and ‘prefer not to say’ options will also be included]

- Antibiotics
- Antihistamines

- Antitussives
- Bronchodilators
- Expectorants
- Honey
- Mucolytics
- Steroids (nasal/inhaled)
- Steroids (systemic)
- Proton pump inhibitors
- Immunostimulants
- Leukotriene receptor antagonists

Q3.16

[Ask all]

Under what circumstances do you recommend using antibiotics for pediatric cough?

[Open-ended]

Q3.18

[Ask all]

In the past 6 months, have you recommended a combination of treatments for any pediatric patients with cough?

[Select one]

- Yes (please specify) [additional textbox]
- No
- Don’t remember
- Prefer not to say

Q3.17

[Ask all]

In the past 6 months, have you recommended phytomedicines for the treatment of cough in any of your pediatric patients?

[Select one]

- Yes [go to Q3.17b]
- No
- Don’t remember
- Prefer not to say

Q3.17b

[Ask if answered 'yes' to Q3.17]

Which phytomedicine formulations/products did you recommend that the pediatric patient(s) use, and why?

[Open-ended]

4. Pediatric cough – unmet needs

In this short section, we will ask for your opinion on unmet needs in pediatric cough.

Q4.1

[Ask all]

What, in your opinion, are the greatest unmet needs in pediatric cough?

[Open-ended]

Q4.2

[Ask all]

What data gaps are there in pediatric cough research that you would like to see addressed in a clinical study?

[Open-ended]

5. Prospan® for pediatric cough

[This section of the questionnaire has been removed since it will be presented in a companion article.]

6. End

Thank you for participating in this survey. We greatly value your contribution.
